# First child’s impact on parental relationship: an existential perspective

**DOI:** 10.1186/s12884-018-1802-5

**Published:** 2018-05-10

**Authors:** Christina Prinds, Ole Mogensen, Niels Christian Hvidt, Mette Bliddal

**Affiliations:** 1 Department of Clinical Institute, University of Southern Denmark, Kløvervænget 10, 5000 Odense C, Denmark; 2 University College South Denmark, Degnevej 16, 6705 Esbjerg Ø, Denmark; 3 Departmen of Gynaecology and Karolinska Institute, Karolinska University Hospital, 17176 Stockholm, Sweden; 4 University of Southern Denmark, Institute of Public Health, J.B. Winsløws Vej 9, DK-5000 Odense, C, Denmark; 5 OPEN – Odense Patient data Explorative Network, Odense University Hospital, J.B Winsløws Vej 9, DK-5000 Odense C, Denmark

**Keywords:** Existential meaning-making, Motherhood, Parental relationship, Partner, Childbirth, Transition

## Abstract

**Background:**

The first child’s birth is for most mothers a profound experience carrying the potential to change life orientations and values. However, little is known of how becoming a mother influences the existential dimensions of life within the parental relationship for example how motherhood may change how we view our partner and what we find important. The aim of this study was to explore how becoming a mother might change the parental relationship seen from the mother’s perspective with a specific focus on dimensions related to existential meaning-making.

**Methods:**

In 2011, 499 Danish first time mothers answered a questionnaire, from which five core items related to changes in the partner relationship from the perspective of the mother, informed this study. The cohort consisted of mothers who gave birth before the 32nd week of gestation (*n* = 127) and mothers who gave birth at full term (*n* = 372). Item 1 focused on thoughts and conversations with her partner about the life change. Item 2 referred to the potential feeling of stronger ties to the partner. Item 3 related to the feeling of being connected to ‘something bigger than one self’ together with the partner. Item 4 focused on potential conflicts due to having a child, and item 5 referred to the experience of dreams. Possible answers ranged from ‘To a high degree’ to ‘Not at all’.

**Results:**

Most respondents found birth of the first child to have forged stronger ties to their partner and have led to both thoughts and conversations about how life together as a couple changed. At the same time, some experienced more conflicts with their partner than before giving birth, however, the majority did actually not. More than half felt their relationship linked to ‘something bigger than themselves’ or had had dreams on being a family.

**Conclusion:**

Findings suggest motherhood transition to be a significant transformer of partnership relation influencing also existential meaning-making. Having the potential to be of importance for the health and vitality of the mother, partner and child, it seems essential to scientifically and clinically address concerns related to existential meaning-making in partner relationship.

## Background

Parenthood is a powerful life-event that often changes life orientations and sometimes increases conflicts among couples [1–3]. The current literature is sparse but a literature review focusing on parenthood and subjective well-being in life finds most cross-sectional and longitudinal evidence to suggest that couples may be happier without children. However, rather than happiness rewards of parenthood the potential reward lies in meaningfulness [4]. The parental relationship is complex due to influences of culture, law, gender, and spirituality. For example, motherhood is something you ‘are’ and this may be perceived differently from fatherhood if fathering is seen as something you ‘do’ rather than something you ‘are’ [1]. The perception of parenthood may also be influenced by deeply rooted considerations of meaning-making in life. However, different explorations on how motherhood and fatherhood are influenced by considerations at an existential level have only been explored briefly. In this study, we focus on the maternal aspect.

Motherhood actualizes considerations related to existential meaning-making. We previously explored existential meaning-making among new first time mothers, who gave birth either at full term or preterm. Surprisingly we found no differences between mothers related to time of birth, when exploring either secular existential concerns or even prayer and meditation practices: Overall, we found mothers in a secular culture like the Danish to experience intensified considerations of death, vulnerability of life, and responsibility regardless of the child was born preterm or at full term [5]. We then explored if motherhood transition actualized prayer and meditation practices, as it is found to be the situation among other Danish hospitalized patients and relatives [6]. We found 65% of the respondents to practise prayer and/or meditation in the postnatal period (6-18 months postpartum), which was higher than among hospitalized patients [7]. Thus, both the significance of certain meaning-making ways and practises related to existential dimensions of human life are intensified during early motherhood [8]. That is interesting findings in a secular context, where public religiosity and shared beliefs diminish, but where religious beliefs nonetheless seem to increase, when becoming parents [9]. If making meaning of life is challenged when becoming a mother, the relationship to the partner might be challenged as well. Therefore the aim of the study was to explore how motherhood might change the partner relationship, focusing on existential meaning-making. We also aimed to explore any potential differences between responses given by mothers who gave birth at full term or preterm.

## Methods

The aim of this study was to explore how becoming a mother changed the parental relationship seen from the mother’s perspective, with a specific focus on dimensions related to existential meaning-making.

This study drew on data collected in 2011 from a national questionnaire survey among Danish first time mothers who gave birth in 2010. Initially, the cohort was sampled from the Danish Medical Birth Registry to investigate differences between full term mothers (> 37 completed weeks of gestation: FT mothers) and mothers giving birth preterm (< 32 completed weeks of gestation: PT mothers). The sampling ratio was 3:1 in favour of FT mothers and PT mothers were therefore over-represented compared to the background population. Both groups included mothers who experienced perinatal loss in the pregnancy for which they were included (stillbirth after 22nd week of gestation or death within 7 days postpartum).

The overall aim of the questionnaire was to measure dimensions of existential meaning-making related to motherhood transition. We used the theoretical framework developed by la Cour and Hvidt to distinguish between *secular, religious,* and *spiritual* ways of making meaning, since existential meaning-making in Northern Europe is considered linked to mainly secular viewpoints, contrary to for example America [8]. We refer to prior work for detailed information about sampling, theoretical framework, scale construction, and survey development [5]. The questionnaire was sent out to 913 mothers during 2011, and since all mothers who gave birth preterm (< 32 completed weeks of gestation) during 2010 were included, the timespan for inclusion varied from 6 to 18 months after childbirth. The response rate was 57% (*N* = 517). We excluded women who reported being without a partner at the time of birth (*N* = 18). The final study population consisted of 499 women. The Regional Research Ethics Committee of Southern Denmark approved the study, and all recommendations from Danish Data Protection Agency were followed.

### The five core items

The questionnaire comprised 46 overall items categorized into seven sections covering aspects of existential meaning-making in early motherhood related to pregnancy, childbirth, and the first 6-18 months postpartum. We focused on one battery consisting of five items designed to provide knowledge about perceptions of partner relationship related to *secular* orientations in existential meaning-making (Table 1). Item 1 focused on how motherhood transition gave rise to both the informants’ owns inner dialogue and her outer dialogue with her partner about the life change. Item 2 referred to the potential feeling of stronger ties to the partner. Item 3 was related to the feeling of somehow being connected to ‘something bigger than one self’, together with the partner. Item 4 focused on potential conflicts due to having a child, and item 5 referred to the experience of dreams (both bad and good dreams), that led to reflections on the changed dynamics in the relationship. These five core items were designed to measure changes in the partner relationship from the perspective of first time mothers in a secular context. The items could be answered with one of the following statements: to a high degree, to some degree, to a small degree, not at all, and don’t know.

**Table 1:** Questions on perceptions on partner relationship related to existential meaning-making in first time new mothers

Overall question	How did becoming a mother change your relationship to your partner? *Assess the statements listed below, and mark with a cross what you think matches you.*
Item 1	Having a baby gave rise to thoughts and conversations between my partner and me about how it has changed our life together
Item 2	Having a baby has forged stronger ties between my partner and me
Item 3	Having a baby has linked our relationship to something bigger than ourselves
Item 4	Having a baby has given rise to more conflicts between me and my partner
Item 5	Having dreams that made me reflect on myself and on us as a family

Items could be answered with to a high degree, to some degree, to a small degree, not at all, and don’t know

### Other data

Data on education was self-reported and categorized as none, short, middle, or long, explained by years of education and exemplified in the questionnaire as e.g. ‘middle (3-4 years, for example bachelor degree)’. We linked data on each woman in the cohort to the Danish Medical Birth Registry by use of the unique individual person identification number assigned to all Danish individuals [10]. The Danish Medical Birth Registry informed on maternal age at birth (continuous), delivery mode (cesarean section: yes/no), if the mother had experienced perinatal/postnatal loss (yes/no), and gestational age, which was categorized as preterm and full term as defined above.

### Statistical analysis

We tabulated the characteristics of the cohort in regard to socioeconomic and obstetric factors. The distribution of answers for each item was graphically displayed and we present proportions of mothers agreeing (by answering ‘to a high degree’ or ‘to some degree’) to each item. Due to the initial selection of the study sample, we used logistic regression models to estimate odds ratios for the association between gestational age (preterm or full term) and agreement with statement in each item. Women giving birth at full term served as reference. Women reporting ‘don’t know’ or did not answer the specific item were excluded in the comparative analyses. In the multivariate analyses, we adjusted for caesarian section and perinatal loss. Results are presented as point estimates with 95% confidence intervals (95% CI). Analyses were performed using STATA 14.0 [11].

## Results

The average age was 29 years and all categories of educational status were represented although few had no education. The median gestational age was 39 weeks (interquartile range 32-41), and 127 (25.5%) gave birth preterm. More than one in four (28%) gave birth by caesarian section, and 32 women (6%) experienced perinatal loss (Table 2). Differences in age and educational status were minor between mothers who gave birth full term or preterm. However, in regard to obstetric factors, PT mothers were more likely to experience caesarean section or perinatal loss.

**Table 2:** Characteristics of the study population according to gestational age (preterm or full term births)

	All	
Study population, *N* (%)	499	(100.0)
Socio-economic factors		
Age at delivery, median (IQR), y	29.0	(27.0-32.0)
Educational status		
None, *N* (%)	45	(9.0)
Short, *N* (%)	160	(32.1)
Middle, *N* (%)	155	(31.1)
Long, *N* (%)	116	(23.2)
Missing, *N* (%)	23	(4.6)
Obstetric factors		
Preterm born, *N* (%)	127	(25.5)
Caesarean section, *N* (%)	141	(28.3)
Perinatal/postnatal loss, *N* (%)	32	(6.4)

*IQR* Interquartile range, *y* years, *wk.* weeks

**Table 3:** Proportion of mothers agreeing to items on existential meaning-making in partner relationship

	Numbers	Agree^a^	Per cent
Thoughts and conversations	478	409	85.6
Forged stronger ties	474	422	89.0
Linked us to something bigger than ourselves	437	261	59.7
Gave rise to more conflicts	473	205	43.3
Dreams leading to reflections	410	255	62.2

^a^Agreed to statement in item

**Table 4:** Crude and adjusted odds ratios for agreeing to item statement according to time of delivery

	Numbers	Agreed^a^	Per cent	OR Crude	(95% CI)	OR Adjust^b^	(95% CI)
Thoughts and conversations							
Full term	360	303	84.2	1.0 (Ref.)	–	1.0 (Ref.)	–
Pre-term	118	106	89.8	1.66	(0.86-3.22)	1.63	(0.79-3.35)
Forged stronger ties							
Full term	357	312	87.4	1.0 (Ref.)	–	1.0 (Ref.)	–
Pre-term	117	110	94.0	2.27	(0.99-5.17)	1.83	(0.77-4.32)
Linked us to something bigger than ourselves							
Full term	330	196	59.4	1.0 (Ref.)	–	1.0 (Ref.)	–
Pre-term	107	65	60.7	1.06	(0.68-1.65)	0.96	(0.58-1.59)
Gave rise to more conflicts							
Full term	359	157	43.7	1.0 (Ref.)	–	1.0 (Ref.)	–
Pre-term	114	48	42.1	0.94	(0.61-1.43)	1.14	(0.70-1.84)
Dreams leading to reflections							
Full term	305	188	61.6	1.0 (Ref.)	–	1.0 (Ref.)	–
Pre-term	105	67	63.8	1.10	(0.69-1.74)	0.84	(0.50-1.41)

^a^Agreed to statement in item

^b^Adjusted for delivery mode and perinatal loss

Overall, we found mothers willing to reply to the five items (non-respondents ranged between 3.8 and 4.6%), and the respondents did also know *what* to reply although item 3 and 5 caused more difficulties in *what* to reply, as respectively 8 and 13% implied they did not know what to answer.

**Fig. 1:**
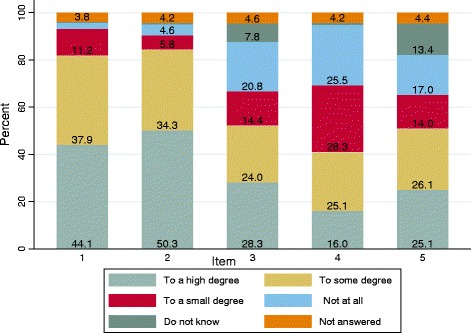
Distribution of answers about perceptions of partner relationship related to secular orientations in existential meaning- making. 1: Talks of change in life together, 2: Forged stronger ties, 3: Linked our relationship to something bigger than ourselves, 4: Gave rise to more conflicts, and 5: Dreams that made me reflect on myself and on us as a family. Numbers are percent, (percentages below 2 not written)

More than 89% of the mothers (Table 3) found that having a child forged stronger ties between themselves and their partner (50% responded to a high degree, Fig. 1). More than 85% found that having a child gave rise to thoughts and conversations with their partner on how it changed their life together. Despite the feeling of stronger ties, 43% experienced more partner conflicts as well. Yet, the item focusing on the potential rise of conflicts was also the one prompting most active disagreements, hence 53% reported not to have experienced more conflicts.

In the two items prompting most ‘I don’t know’- answers (item 3 and 5), 59% found that having a child linked them and their partner to something greater than themselves, and 62% of the mothers found themselves having had dreams facilitating reflections on self and family.

For all items, we found no difference in agreeing to the item statement between mothers giving birth preterm compared to mothers giving birth full term. However, there were small differences in answers tending to more mothers giving birth preterm agreeing to items 1 and 2, whereas item 3-5 were more equally answered among FT and PT mothers (Table 4).

## Discussion

Danish first-time mothers, asked between 6 and 18 months after birth, experienced stronger ties to their partner, which led to both thoughts and conversations about how life together changed. At the same time, a minority (43%) experienced more conflicts than before giving birth. There were no differences in distribution of answers between mothers having given birth full term and preterm. Thus, according to these findings experiencing conflicts in a parental relationship does not necessarily contrast forging stronger ties, which may be an important message to new parents. In general, our results echo other findings pointing to parenthood as both challenge and joy in a relationship [1, 12, 13]. Nelson et al. points to the increase of sleeplessness and more economic worries among parents than non-parents. At the same time parenthood also leads to an increase in wellbeing, probably because of the feeling of meaningfulness and the fulfilment of basic emotional needs [14].

The two items pointing to something subconscious or transcending oneself appeared more difficult to grasp, as more respondents did not know what to answer. These two items were concerned with the feeling of being connected to ‘something bigger than ourselves’ or to have ‘had dreams leading to reflections on self and family’. However, having a child made 59% of the mothers link their relationship to ‘something bigger’ than themselves. As discussed in a previous study, exploring existential meaning-making among first-time mothers, ‘something bigger’ can be interpreted in several ways [5]. It may refer to the feeling of being part of a bigger relational unit including a child or it could be the feeling that the existential fundamentals of life with their partner changed. For some mothers it may refer to the feeling of the relationship being connected to a transcendent dimension [9]. The individually interpreted meaning of the item cannot be explored through this survey. However, the findings suggest that first time mothers find making meaning of life with regards to their partner altered, also when living in a secular society where concerns related to existential meaning-making of both secular, religious, and spiritual are individualised and rarely explicated [15, 16].

More than 62% of the mothers had dreams ‘leading to reflections on self and family’. The heightened level of dreams among pregnant and new mothers are often linked to normal emotional concerns or mood, interpreted as normal but sleep disturbing [17]. However, the use of dream-themes in clinical practice to address basic existential concerns in this period of life seem scientifically unexplored, although matters of life, death and responsibility seem brought to the forefront [5, 18].

Previously factors such as couple communications skills, child temperament, and sleep patterns have been found to change in relation to the specific event of couples’ transition to parenthood [3, 19]. However, findings from this study indicate, that changes in existential meaning-making for example in feeling connected to ‘something bigger than ourselves’ might also be important since more than half of the respondents agreed to it. The bare awareness and acknowledgement of existential dimensions of relationship-changes might be of value although we do not know the deeper meaning of each mother’s response to this item [9]. Dimensions of existential meaning-making may also impact mental health, including anxiety and depressive symptoms, which have been found to be predictive in relationship decline [13].

Two underlying basic assumptions grounded the study: firstly, that pregnancy, birthing and the postpartum period both individually and as a complete experience can facilitate considerations of existential meaning, and therefore change partner relationship. Connected to this, we also assumed answers were different between FT and PT mothers, since research among mothers having given birth preterm underline this as a very stressful period [20, 21]. In the analyses of mothers giving birth preterm or full term and agreeing to the statements, we found only minor differences in answers from FT and PT mothers respectively in their perception of change in partner relationship after adjustment for perinatal loss and mode of delivery. The lack of difference in answers indicates motherhood transition itself, independent from time of birth, to facilitate changes in existential meaning-making, which again facilitates changes in partner relationship. This is also in concordance with our earlier research [5]. However, being the most profound experience in many women’s lives, it remains scientifically unexplored, how motherhood changes existential meaning in life – and thereby partnership relations, although focusing on familial and relational aspects is considered important for health in WHOs recommendations [22].

In other arenas of health care services, for example in the field of palliation, care of existential or spiritual concerns in life is sought embedded, in line with recommendations from WHO and national authorities [23, 24]. Our results suggest that it may also be vital to further explore dimensions of existential concerns in life including the change of partnership relation after having the first child, especially since research and interventions in maternity care points to the challenges in preserving healthy relationships after childbirth [3, 25].

### Strengths and limitations

Among the strengths in this study is the use of a transparent theoretical framework guiding the development of the survey, thereby enabling cultural sensitive exploration of existential meaning-making in relation to motherhood transition [8, 26]. No prior survey has previously phrased questions on postpartum relationship addressing existential meaning-making. Despite the comprehensive questionnaire, the response rate was higher than in comparable surveys addressing items related to values and existential life views [27, 28].

We are well aware, that our findings rely on 5 items exploring partnership relation on a five point Likert scale, which lacks nuances and complexity. However it indicates directions of how partnership relations may evolve when becoming a mother. Furthermore findings relates to the period between 6 and 18 months postpartum. The wide timespan may affect responses leading to different perceptions of partner relationship between mothers who gave birth 6 months previously and those who gave birth 18 months previously. Research suggests relationship satisfaction to decline over a 3-year period from second trimester to 30 months postpartum [13] and our results may have been different if the survey had been performed in another time frame.

## Conclusion

Through the lens of a first-time mother in a secular society, having a child forged stronger ties to her partner and increased thoughts and conversations of the life change with the partner. Less than half experienced more conflicts with their partner 6-18 months postpartum. More than half felt their relationship linked to ‘something bigger than ourselves’ and had dreams facilitating reflections on family and selves. Although this cross-sectional survey does not leave room for interpretational complexity, findings suggest motherhood transition to be a potential transformer of partnership relation with regard to existential meaning-making. Future research should provide in-depth exploration in this field with the use of qualitative methods. Moreover, in the light of these findings, we suggest research to focus also on existential meaning-making through the lens of fatherhood transition, since becoming a father may also potentially also transform partnership relation. Having the potential to be of importance for the health and vitality of the mother, partner, and child, it seem essential to scientifically and clinically address concerns related to existential meaning-making in partner relationship.
